# Gadolinium-DTPA: A New Contrast Agent

**Published:** 1988-05

**Authors:** H. P. Niendorf

**Affiliations:** Schering AG, Berlin, FRG


### Gadolinium-DTPA: A New Contrast Agent

**Dr H. P. Niendorf,** Schering AG, Berlin, FRG

Gadolinium (Gd)-DTPA-dimeglumine (tradename: Magnevist®) is the first commercially available contrast agent for magnetic resonance tomography (MRT). As a result of the very favourable results from preclinical and clinical investigations Magnevist® was granted a product license for cranial and spinal MRT in the Federal Republic of Germany in early February this year.

Magnevist® is a paramagnetic, water soluble and well tolerated contrast agent. Following intravenous injection Magnevist® is rapidly distributed in the intravascular and extravascular extra-cellular space with a distribution half-life of 12 minutes. It is excreted by glomerular filtration with an elimination half-life of 90 minutes. 91 (±13) % of the given dose is excreted within the first 24 hours following application. Magnevist® does not pass the unimpaired blood-brain barrier. In short, the pharmacokinetic behaviour of Magnevist® is similar to that of the well-known iodinated urographic contrast agents (1).

The excellent tolerance and safety of Magnevist® have been shown in more than 5800 patients examined with doses of either 0.1 or 0.2 mmol Magnevist® per kg body weight. Available safety data suggest that Magnevist® may well turn out to be even better tolerated than the modern non-ionic x-ray contrast media. The recommended clinical dose is 0.1 mmol/kg body weight (2).

It is worth mentioning that Magnevist® itself does not produce signals but rather influences the signal producing properties (T1 and T2) of hydrogen atoms, mainly T1. Within the recommended dose range (0.1 mmol/kg to 0.2 mmol/kg) and using T1-weighted sequences the shortening of T1 will result in a net increase of signal intensity (SI) which in turn, in most instances, will lead to a diagnostically useful image contrast enhancement.

The diagnostic usefulness of Magnevist® has been reported in numerous publications.

In general, Magnevist® significantly increase the sensitivity and specificity of MRT. Diagnostic advances of Magnevist® are best appreciated in indications in which unenhanced MRT alone has been found to successfully challenge other imaging techniques such as x-ray CT. Magnevist® also facilitates the use of fast MR imaging techniques, such as FLASH and others, making up for loss in image quality. In addition, it enables dynamic studies for the assessment of organ and pathology perfusion and/or function (kidney, liver, brain tumours etc.).

The most thoroughly investigated area of use of Magnevist® in MRT is the central nervous system. Amongst other things, Magnevist® has been found to be especially useful in increasing tumour detection rate (metastases, small tumours) and in improving tumor classification. The latter by allowing the differentiation of vital tumour tissue (well perfused and/or with impaired blood-brain barrier) from central necrosis and from surrounding edema or uninvolved tissue. This pattern of tumour enhancement is very similar to that of contrast-enhanced CT, which allows the transfer and full use of the radiologist's experience in the interpretation of the images.

The contrast enhancing effect of Magnevist® combined with the ease of demonstrating a lesion in different planes (sagittal, coronal, transversal) with MRT has proven to be of great use in pre-operative planning.

It seems very likely that Magnevist® will play a major role in MRT, by facilitating the differentiation of acute from chronic lesions in multiple sclerosis, and also by helping to differentiate acute myeloencephalitis from multiple sclerosis.

In MRT of the spine Magnevist® has been found to be especially useful. For example, in the differentiation of tumourcyst with adjacent enhancing tumour tissue from syrinx, and the detection of leptomeningeal disease of the spine. Also Magnevist® is of use in cases of suspected recurrent disc disease.

One of the most promising indications outside the CNS may well be the detection and delineation of inflammatory soft tissue disease and of paraosseus tumourous and/or edematous involvement in tumours of the skeletal system. As a result Magnevist® helps to improve local tumour staging and monitoring of pre-operative cytostatic therapy response (3-5).

MR imaging of the female breast with Magnevist® seems to hold promise for further diagnostic information in cases where mammography and ultrasound have failed to furnish sufficient information (6-9).

In all above described indications Magnevist® is of special value when relapse of a known condition is suspected and further information is needed from MRT.

The role of Magnevist® in MRT has yet to be fully assessed. Existing indications are being probed and new indications are being developed. At this point the investigation of Magnevist® for MR arthrography has been initiated and an oral formulation of Gd-DTPA is being developed as a marker of the GI-tract. The introduction of Magnevist® into clinical routine MRT is bound to bring about further indications for its useful application which will benefit patients and doctors.

### REFERENCES

1. WEINMANN, H. J., LANIADO, M., MÜTZEL, W. (1984) Pharmacokinetics of Gd-DTPA/Dimeglumine after intravenous injection. *Physiol Chem Physics Med NMR* **16**, 167–172
2. NIENDORF, H. P., LANIADO, M., SEMMLER, W. *et al.* (1987) Dose administration of Gd-DTPA in MR imaging of intracranial tumours *AJNR* **8**, 803–815.
3. REISER, M., KAHN, T., RUPP, N. *et al.* (1986) Ergebnisse der MR Tomographie in der Diagnostik der Osteomyelitis und Arthritis *Fortsch Röntgenstr* **145**, 661–666.
4. REISER, N., BOHNDORF, K., NIENDORF, H. P. *et al.* (1987) Erste Erfahrungen mit Gd-DTPA in der MR von Knochen- und Weichteilturmoren *Radiologe.* **27**, 467–472.
5. REISER, M., ERHARD, W., BAUER, R. *et al.*(1986) Gd-DTPA-enhanced magnetic resonance imaging in the diagnosis of inflammatory and neo-plastic musculoskeletal lesions. In RUNGE, V. M., CLAUSSEN, C., FELIX, R., JAMES, A. E. (Eds). Contrast Agents in Magnetic Resonance Imaging, Excerpta Medica, Amsterdam pp. 170–176.
6. BECK, R., HEYWANG, S., EIERMANN, W. *et al.* (1987) Konstrastmittelaufnahme der Mamille bei Kernspintomographie der Mamma mit Gd-DTPA *Digit Bilddiagn.* **7**, 167–169.
7. HEYWANG, S., FENZL, G., BECK R. *et al.* (1986) Anwendung von Gd-DTPA bei der kernspintomographischen Untersuchung der Mamma *Fortschr Röntgenstr.* **145**, 565–571.
8. HEYWANG, S., HAHN, D., SCHMIDT, H. *et al.* (1986) MR imaging of the breast using gadolinium-DTPA. *JCAT* **10**, 199–204.
9. HEYWANG, S., FENZL, G., EIERMANN, W. *et al.* (1989) Magnetic resonance imaging of the breast with Gd-DTPA; In: RUNGE, V. M., CLAUSSEN, C., FELIX, R., JAMES, A. E. (Eds.), Contrast Agents in Magnetic Resonance Imaging, Excerpta Medica, Amsterdam pp 155–158.

